# Inhibition of Casein Kinase 2 Protects Oligodendrocytes From Excitotoxicity by Attenuating JNK/p53 Signaling Cascade

**DOI:** 10.3389/fnmol.2018.00333

**Published:** 2018-09-13

**Authors:** Manuel Canedo-Antelo, Mari Paz Serrano, Andrea Manterola, Asier Ruiz, Francisco Llavero, Susana Mato, José Luis Zugaza, Fernando Pérez-Cerdá, Carlos Matute, María Victoria Sánchez-Gómez

**Affiliations:** ^1^Achucarro Basque Center for Neuroscience, Leioa, Spain; ^2^Departamento de Neurociencias, Universidad del País Vasco (UPV/EHU), Leioa, Spain; ^3^Centro de Investigación en Red de Enfermedades Neurodegenerativas (CIBERNED), Leioa, Spain; ^4^Departamento de Genética, Antropología Física y Fisiología Animal, Universidad del País Vasco (UPV/EHU), Leioa, Spain; ^5^IKERBASQUE, Basque Foundation for Science, Bilbao, Spain

**Keywords:** CK2, AMPA receptor, oligodendrocyte, apoptosis, mitogen-activated protein kinase (MAPK), c-Jun N-terminal kinase (JNK), p53, excitotoxicity

## Abstract

Oligodendrocytes are highly vulnerable to glutamate excitotoxicity, a central mechanism involved in tissue damage in Multiple Sclerosis (MS). Sustained activation of AMPA receptors in rat oligodendrocytes induces cytosolic calcium overload, mitochondrial depolarization, increase of reactive oxygen species, and activation of intracelular pathways resulting in apoptotic cell death. Although many signals driven by excitotoxicity have been identified, some of the key players are still under investigation. Casein kinase 2 (CK2) is a serine/threonine kinase, constitutively expressed in all eukaryotic tissues, involved in cell proliferation, malignant transformation and apoptosis. In this study, we identify CK2 as a critical regulator of oligodendrocytic death pathways and elucidate its role as a signal inductor following excitotoxic insults. We provide evidence that CK2 activity is up-regulated in AMPA-treated oligodendrocytes and CK2 inhibition significantly diminished AMPA receptor-induced oligodendroglial death. In addition, we analyzed mitogen-activated protein kinase (MAPK) signaling after excitotoxic insult. We observed that AMPA receptor activation induced a rapid increase in c-Jun N-terminal kinase (JNK) and p38 phosphorylation that was reduced after CK2 inhibition. Moreover, blocking their phosphorylation, we enhanced oligodendrocyte survival after excitotoxic insult. Finally, we observed that the tumor suppressor p53 is activated during AMPA receptor-induced cell death and, interestingly, down-regulated by JNK or CK2 inhibition. Together, these data indicate that the increase in CK2 activity induced by excitotoxic insults regulates MAPKs, triggers p53 activation and mediates subsequent oligodendroglial loss. Therefore, targeting CK2 may be a useful strategy to prevent oligodendrocyte death in MS and other diseases involving central nervous system (CNS) white matter.

## Introduction

Glutamate excitotoxic cell death can occur in virtually all cells that express ionotropic glutamate receptors (GluRs) including oligodendrocytes (Yoshioka et al., 1995; Matute et al., 1997; McDonald et al., 1998) and it has been implicated in acute injury to the central nervous system (CNS) and in chronic neurodegenerative disorders (Lipton and Rosenberg, 1994; Choi, 1988; Lee et al., 1999). Oligodendrocytes are particularly vulnerable to glutamate insults through AMPA and kainate receptor-mediated excitotoxicity, a central feature in the pathogenesis of demyelinating disorders such as Multiple Sclerosis (MS; Matute et al., 2007).

Excitotoxic insults to oligodendrocytes are dependent on Ca^2+^ entry through ionotropic GluRs, which alters Ca^2+^ homeostasis and induces changes in the mitochondrial membrane. This mitochondrial dysfunction is mainly mediated by Bax (Sánchez-Gómez et al., 2011; Simonishvili et al., 2013), a proapoptotic member of the Bcl-2 protein, and leads to cytochrome *c* release to the cytosol, where it can activate caspase-9 and downstream caspase-3 and trigger apoptosis (Galluzzi et al., 2009). However, additional proapoptotic signaling pathways initiated by AMPA receptors upstream to mitochondrial dysfunction are relatively unexplored and the involvement of certain molecules that potentially contribute to or oppose the apoptotic cascade still remain unknown.

Protein Casein Kinase 2 (CK2) is a highly conserved serine/threonine kinase present in all tissues, eukaryotic cells and most cellular compartments. CK2 can form a tetrameric structure consisting of two α-subunits with catalytic activity, and two β-subunits that regulate enzymatic activity and substrate specificity (Vilk et al., 1999). The first physiological targets of this kinase were detected in the late 1970s to reach the number of more than 300 in the 2,000 s (Meggio and Pinna, 2003) and it is predictable that proteins phosphorylated by CK2 are much more numerous than those identified to date. Attesting to its importance, changes in CK2 activity are usually associated with significant changes in cell fate. Although the overall function of CK2 is not completely understood, CK2 activity has been associated with many cellular processes including cell cycle progression, differentiation, cell migration, polarity establishment and transformation (Litchfield, 2003; Poole et al., 2005). CK2 activity is a potent and multifunctional promoter of cell growth and survival, and because of that it is currently considered a promising target for cancer therapy (Hanif et al., 2010; Pierre et al., 2011). Nonetheless, in contrast to the evidence that CK2 functions as a cell survival mediator, several studies have described a proapoptotic contribution for this enzyme specifically linked to c-Jun N-terminal kinase (JNK) activation (Min et al., 2003; Hilgard et al., 2004). In addition to its apoptotic function, a number of studies have suggested a pro-inflammatory role for CK2, including investigations using experimental autoimmune encephalomyelitis (EAE), a key animal model for MS. These studies established that the CD5-dependent CK2 signaling pathway represents a major signaling cascade initiated by CD5 that regulates the threshold of T cell activation and Th differentiation and impacts the outcome of EAE, so that mice deficient in CD5-CK2 signaling pathway are mostly resistant to EAE (Axtell et al., 2006; Sestero et al., 2012; Mier-Aguilar et al., 2016). In addition, CK2 pharmacological inhibition ameliorates EAE severity and relapse incidence (Ulges et al., 2016) as well as attenuates apoptosis and inflammatory cell infiltration after renal ischemia-reperfusion injury (Ka et al., 2015).

Given that apoptosis and inflammation are critical events for MS, CK2 activation may have some role in the pathogenesis of MS not only restricted to pro-inflammatory events but also in apoptotic cascade induced by concomitant excitotoxic context. However, it is unknown whether CK2 is involved in the vulnerability of oligodendrocytes during excitotoxic insults. In the present study, we investigated the possible role of CK2 in this deleterious process and its potential relationship with other molecular effectors of death.

## Materials and Methods

### Ethics Statement

This study was carried out in accordance with the recommendations and the approval of the internal animal ethics committee of the University of the Basque Country (UPV/EHU), in accordance with the European Communities Council Directive. All the protocols were approved by the “Ethics Committee on Animal Experimentation (CEEA)” which is a collegiate authority into the operational structure of the Ethics Commission for Research and Teaching (CEID) of the University of the Basque Country. The committee CEEA is also authorized by the Ministry of Science and Innovation to evaluate projects that experiment with animals. All possible efforts were made to minimize animal suffering and the number of animals used. Rats and mice in both sexes were used for all experiments.

### Oligodendrocyte Culture

Highly enriched OPCs were prepared from mixed glial cultures obtained from newborn (P0–P2) Sprague–Dawley rat forebrain cortices as previously described (McCarthy and de Vellis, 1980) with minor modifications (Chen et al., 2007; Bernal-Chico et al., 2015). Briefly, forebrains were removed from the skulls and the cortices isolated and enzymatically digested by incubation with 0.25% trypsin and 4% DNAse for 15 min at 37°C. Then, the tissue was mechanically dissociated and plated in Iscove’s Modified Dulbecco’s Medium (IMDM) supplemented with 10% fetal bovine serum (FBS, Hyclone). The mixed glial cells were grown in T75 flasks (pre-treated with poly-d-lysine) until they were confluent (10–12 days). Microglia were separated from the cultures by shaking the flasks on a rotary shaker for 1 h at 250 revolutions/min. OPCs were isolated following an additional 18 h. OPCs were seeded on to poly-d-lysine-coated coverslips and were maintained at 37°C and 5% CO_2_ for 2 days in a chemically defined proliferation medium SATO composed of: 100 μg/ml BSA, 100 μg/ml transferrin, 16 μg/ml putrescine, 60 ng/ml progesterone, 40 ng/ml selenium, 6.3 mg/ml N-acetyl-cysteine, 0.5 mg/ml insulin (all of from Sigma) and growth factors b-FGF (5 ng/ml) and PDGF-AA (5 ng/ml; from Peprotech) to amplify the number of oligodendroglial cells. These conditions produced cultures that contain 95 ± 0.2% Olig2+, 92.5 ± 0.5% PDGF-Rα+ oligodendroglial cells, and 1.4 ± 0.3% GFAP+ astrocytes and 2.4 ± 0.5% Iba1+ microglia. After the proliferation stage, medium was replaced with maturation SATO composed by 100 μg/ml BSA, 100 μg/ml transferrin, 16 μg/ml putrescine, 40 ng/ml thyroxine, 30 ng/ml tri-iodothryronine, 60 ng/ml progesterone, 40 ng/ml selenium, 6.3 mg/ml N-acetyl-cysteine, 0.5 mg/ml insulin (all from Sigma), plus 1 μg/ml CNTF and 10 μg/ml NT3 (both from Peprotech) to induce oligodendrocyte maturation. Cells were used 2 days later for different experiments whose common point is the treatment with 10 μM AMPA plus 100 μM cyclothiazide (CTZ; added 5 min before AMPA) for 30 min to recreate AMPA-induced excitotoxic conditions (Sánchez-Gómez and Matute, 1999).

### *In vitro* CK2 Kinase Assay

Oligodendrocytes were incubated in the absence or presence of CK2 inhibitor (TBB; 5 μM for 3 h) or CK2 activator (spermine; 10 μM for 1 h), and then treated with 10 μM AMPA plus CTZ for 30 min. Thereafter, cells were washed twice with cold phosphate-buffered saline (PBS), lysed with lysis buffer (20 mM Tris, pH 7.4, 137 mM NaCl, 5 mM EDTA, 1 mM EGTA, 10 mM NaF, 1 mM sodium pyrophosphate, 100 μM β-glycerophosphate, 10 μg/ml of aprotinin, 1 mM PMSF, 10% glycerol, and 1% (v/v) Triton X-100) and lysates were clarified by centrifugation at 12,300 *g* for 10 min at 4°C. Supernatants were recollected and subjected to CK2α immunoprecipitation by the addition of CK2α specific antibody (1:500; Santa Cruz Technologies) and the immunocomplex formed were captured by incubation with Gamma Bind Plus-Sepharose beads (GE Healthcare) for 4 h with gentle rotation at 4°C. Then, the mix was centrifuged at 400 *g* for 1 min and the supernatants were discarded. The beads were washed twice with cold lysis buffer, twice with cold washing buffer (10 mM HEPES, pH 7.4, 100 mM NaCl, 20 μg/ml of aprotinin, and 0.5% IGEPAL-360) and resuspended in reaction buffer (20 mM Tris, pH 7.4, 20 mM NaCl, 1 mM DTT, 10 mM MgCl_2_, and 1 mM MnCl_2_). Then, 40 μl of each sample were assayed for *in vitro* kinase reaction in the presence of 500 ng of purified recombinant GST-RCAN3 (generous gift from Dr. Mercé Pérez-Riba), which represent a potential serine phosphorylation target of CK2 (Martínez-Hoyer et al., 2013), and 200 μM ATP. The reaction was carried out for 30 min at 30°C and stopped by adding 30 μl of 2× loading buffer. The samples were boiled and resolved by SDS-PAGE and immunoblotting using a mouse anti-phosphoserine antibody (anti-pSerine; 1:1,000; Sigma Aldrich), a rabbit anti-GST antibody (1:5,000; Abcam) or a rabbit anti-CK2α (1:500; Sta. Cruz Technologies) and enhanced chemiluminescence detection kit, according to the manufacturer’s instructions (Supersignal; Thermo Scientific). In addition, western blotting against CK2α was developed in whole cell lysate to confirm that equivalent amounts of CK2α were present in all samples.

### Quantitative PCR

Total RNA was extracted from cell cultures with TRIzol reagent according to the manufacturer’s recommendations (Invitrogen, Barcelona, Spain). First strand cDNA synthesis was performed with reverse transcriptase SuperScript™ III (Invitrogen) using random primers as previously described (González-Fernández et al., 2014). Specific primers for CK2α, CK2β, MDM2 and PUMA were designed with the PrimerExpress software (Applied Biosystems) and were targeted to exon junctions to avoid amplification of contaminating genomic DNA. Primers sequences were as follows: rat CK2α forward TGGTGGAATGGGGAAATCAAG and reverse CCTGCCTAATTTT CGAACAAGC; rat CK2β forward GGACCTGGAACCTGATGAAGAG and reverse TTTCCAACATTTGTGCAATGC; rat MDM2 forward AGGATGATGAGGTCTATCGGGTC and reverse GAGGATTCATTTCATTGCACGA; rat PUMA forward CAGTGCGCCTTCACTTTGG and reverse GGGTTGAGAAGGCTTTCACATG.

Real-time quantitative PCR (qPCR) was performed using SsoFast™ EvaGreen Supermix (Bio-Rad, Barcelona, Spain). Amplifications were run in a CFX96TM Real Time System (Bio-Rad). Each value was normalized against GADPH and HPRT levels as endogenous reference and relative gene expression data were determined by the 2^−ΔΔCt^ method.

### Toxicity Assays

Toxicity assays were performed as described previously (Sánchez-Gómez et al., 2003), with minor modifications. Oligodendrocytes, after 2 days in maturation medium, were exposed to 100 μM CTZ for 5 min before incubation with 10 μM AMPA for 30 min. The medium was subsequently removed and the cells incubated overnight in AMPA-free fresh medium. For studies with the different activator or inhibitors, cells were pretreated with the dilution and for the time indicated for each drug before the incubation with CTZ/AMPA, and all drugs were present during stimulus application. These compounds used were: CK2 activator spermine; CK2 inhibitors TBB, DRB and resofurin; JNK inhibitor SP600125; p38 inhibitor PH79804 and p53 inhibitors pifithrin-α and pifithrin-μ. Oligodendrocyte viability was assessed 24 h later by loading cells with 1 μM calcein-AM (Molecular Probes; Invitrogen; C3100) for 30 min and fluorescence was measured using a Synergy-HT fluorimeter (Bio-Tek Instruments Inc., Winooski, VT, USA) as indicated by the supplier (485 nm excitation wavelength and 530 nm emission wavelength). All experiments were performed in duplicate, and the values provided here are the averages of at least three independent experiments.

### Immunocytochemistry

Oligodendrocytes were obtained from mixed glial cultures and treated for AMPA receptor activation as described above. Following treatments, cells were rinsed in PBS and fixed in 4% paraformaldehyde in PBS for 20 min at room temperature, permeabilized in 0.1% Triton X-100 in PBS and processed for immunofluorescence with rabbit anti-CK2α (1:100; Santa Cruz Technologies), anti-total p53 (FL-393; 1:100; Santa Cruz Technologies) or anti-phospho-p53 (Ser15; 1:100; Cell Signaling) and anti-COX-IV (1:200; Cell Signaling) in experiments in which mitochondria were stained. Primary antibodies were detected after incubation with anti-rabbit IgG (H+L) Alexa Fluor-488 or Alexa Fluor-594 (1:200; Invitrogen) secondary antibody, for 2 h at room temperature. Incubation for 10 min with DAPI (1:1,000) was used, when required, to identify nuclei. Cells were washed three times in PBS, mounted on slides with Glycergel (Dako, Glostrup, Denmark), and visualized with a laser scanning confocal microscope (Olympus Fluoview FV500) at the Analytic and High Resolution Microscopy Facility in the University of the Basque Country. No staining was detected in control samples run in parallel without primary antibodies.

### Measurement of Intracellular Reactive Oxygen Species

Accumulation of reactive oxygen species within cells was measured, as previously described (Sánchez-Gómez et al., 2011) by loading cells with 10 μM of the oxidation-sensitive fluorescent dye 5-(and-6)-chloromethyl-2′,7′-dichlorodihydrofluorescein diacetate (CM-DCFDA; Molecular Probes, Invitrogen; C6827), for 20 min at 37°C, 5% CO_2_, using 1 μM calcein-AM as a control dye. Fluorescence was measured using a Synergy-HT fluorimeter (Bio-Tek Instruments Inc., Winooski, VT, USA) and data were expressed as a normalized percentage of CM-DCFDA/calcein fluorescence in controls (untreated oligodendrocytes; 100%). Excitation and emission wavelengths for CM-DCFDA were 488 and 515 nm, respectively. All assays were performed in duplicate and the values are the average of at least three independent experiments (mean ± SEM).

### Analysis of Mitochondrial Membrane Potential

Oligodendrocyte cultures were exposed to AMPA alone or in presence of different drugs and changes in mitochondrial membrane potential were monitored by reduction of 5, 5′,6,6′-tetrachloro-1,1′,3,3′-tetraethylbenzimidazolcarbocyanine iodide (JC-1; Invitrogen), according to the manufacture’s protocol. Briefly, after drug treatment, cells were loaded with 3 μM JC-1 in culture medium for 15 min at 37°C and were washed with HBSS without phenol red two times to eliminate the excess of dye. In the cytosol the monomeric form of this dye fluoresces green (emission read at 529 nm when excited at 485 nm), whereas within the mitochondrial matrix highly concentrated JC-1 forms aggregates fluoresces red (emission at 590 nm when excited at 485 nm). Both JC-1 monomers and aggregates were detectable using a Synergy-HT fluorimeter (Biotek Instruments) and the changes in mitochondrial potential were calculated as the red/green ratio for each condition. The data were expressed as a normalized percentage of fluorescence in controls (untreated cells; 100%). All experiments (*n* ≥ 3) were performed at least in triplicate and plotted as mean ± SEM.

### Western Blotting Analysis

Total protein from rat oligodendrocytes (2 × 10^5^ cells) was obtained at specific time points after the drug treatment by washed with ice-cold PBS and then collected by mechanical scraping with 50 μl of RIPA buffer (50 mM Tris, pH 7.5, 150 mM NaCl, 0.5% Na-desoxycolate, 0.1% SDS, 1% NP-40, 1 mM EDTA) supplemented with Halt™ protease and phosphatase inhibitor cocktail (Thermo Fisher Scientific, Spain). Lysates were boiled in sample buffer for 5 min, and separated by electrophoresis using Criterion TGX Precast 12% gels and transferred to Trans-Blot Turbo Midi PVDF Transfer Packs (Bio-Rad, Hercules, CA, USA). After electroblotting on PVDF membranes (Bio-Rad), these were blocked in 5% skimmed milk, 5% serum in TBST for 1 h. The blots were probed with primary antibodies in 5% BSA in TBST: rabbit anti-CK2α (1:500; Santa Cruz Technologies); rabbit anti-phospho-JNK (1:1,000; Cell Signaling); rabbit anti-JNK (1:1,000; Cell Signaling); rabbit anti-phospho-p38 (1:1,000; Cell Signaling); rabbit anti-p38 (1:1,000; Cell Signaling); mouse anti-total p53 (1C12; 1:500; Cell Signaling); rabbit anti-PUMA (1:1,000; Cell Signaling); rabbit anti-phospho-p53 (Ser15; 1:1,000; Cell Signaling); rabbit anti-actin (1:5,000; Sigma) overnight at 4°C. After washing, the blots were developed using HRP-conjugated anti-IgG (1:5,000) and an enhanced chemiluminescence detection kit, according to the manufacturer’s instructions (Supersignal; Thermo Scientific). Images were acquired with a ChemiDoc MP system (BioRad) and quantified with ImageJ software.

### AMPA Receptor Activation in Isolated Mice Optic Nerves

Transgenic mice PLP-DsRed, generous gift from Dr. Frank Kirchhoff (University of Saarland, Homburg, Germany), were used in which fluorescent reporter DsRed was under control of the glial-specific promoter proteolipid protein (PLP; Hirrlinger et al., 2005). Optic nerves from P25 PLP-DsRed mice were dissected out in cold oxygen-saturated artificial CSF containing 126 mM NaCl, 3 mM KCl, 2 mM MgSO_4_, 26 mM NaHCO_3_, 1.25 mM NaH_2_PO_4_, 2 mM CaCl_2_ and 10 mM glucose and were freed from of their meninges, as previously described (Sánchez-Gómez et al., 2003). Subsequently, nerves were placed in a 48-well plate containing artificial CSF and pre-incubated with 25 μM TBB for 3 h or 20 μM SP600125 for 1 h, at 37°C and 5% CO_2_, and then treated with 100 μM CTZ for 15 min before addition of AMPA (100 μM; 2 h). Following incubation, media was replaced with fresh oxygen-saturated artificial CSF and incubated for another 90 min at 37°C. Tissue damage was analyzed by measuring lactate dehydrogenase (LDH) release in the incubation media at 30, 60 and 90 min post-stimulus, using the CytoTox 96^®^ assay (Promega Biotech Ibérica, Spain). At the end of LDH evaluation time course and to monitor tissue damage by histology, optic nerves were fixed for 1 h in 4% paraformaldehyde, washed in PBS and mounted on glass slides with ProlLong^®^ Gold antifade mounting media (Invitrogen; Molecular Probes) and seales with coverslips. Images were acquired using a laser scanning confocal microscope (Olympus Fluoview FV500) at the Analytic and High Resolution Microscopy Facility in the University of the Basque Country, with a 10× objective. The number of z-sections were optimized with FluoView software, sections were <0.75 μm thickness, and approximately 15 z-sections for each optic nerve, until analyzing it completely. The fluorescence signal corresponding to the PLP-DsRed^+^ oligodendrocytes that were present in the images was quantified by ImageJ software and the data were expressed as arbitrary units of fluorescence for each experimental situation.

Cell counts of PLP-DsRed^+^ oligodendrocytes in intact optic nerves were performed on flattened confocal z-stacks of 15 μm thickness, taken with a 20× objective along the entire nerve, with a field of view (FOV) volume of 6 × 10^6^ μm^3^ (based in Azim and Butt, 2011). Cells expressing DsRed fluorochrome within this area were counted using the ImageJ “Cell Counter” plugin and the results were represented as mean ± SEM cells per FOV (*n* ≥ 4), where* n* represents number of mice in each experimental condition.

To analyze oligodendrocyte cell death, immunohistochemistry was performed and damage was determined by imaging and DAPI labeling. After treatments, nerves were fixed for 2 h in 4% PFA, in 0.1 M sodium phosphate buffer, cryoprotected in 15% sucrose and frozen. Cryostat sections (10 μm) were mounted onto glass slides and processed for immunofluorescence with rabbit antibodies to APC (Ab-7; 1:200, Calbiochem), specific marker for oligodendrocytes, and fluorescein-conjugated anti-rabbit (1:200; Alexa Fluor 488; Molecular Probes). The sections were incubated with DAPI (1:1,000) for nuclear staining and processed for viewing under confocal microscope as indicated above.

### Gene Silencing Procedures

SureSilencing shRNA Plasmids (Sh-CK2; Csnk2a1 SureSilencing shRNA Plasmid (KR43385) and control negative Sh-Scrambled; Qiagen) were first amplified into competent *E. coli* cells. Growing colonies were picked for large-scale bacterial culture and then plasmids were purified using Maxiprep Kit (PureLink^®^HiPure Plasmid Filter Maxiprep Kit; Invitrogen).

Rat oligodendrocytes (1.5 × 10^6^ cells) were transfected in suspension with gene-specific (Sh-CK2α) and negative control (Sh-Scrambled) shRNA plasmids, both of them including a puromycin resistence gene. Transfection was carried out using Rat Glial Nucleofector Kit (Lonza, Basel, Switzerland) according to the manufacturer instructions, and cells were immediately plated and maintained in maturation medium, as described above. Following transfection, cells were replated 16–18 h into SATO medium containing puromycin (0.3 μg/ml) to begin the process of selecting for and cloning stably transfected cells. One day after the withdrawal of the puromycin, transfected oligodendrocytes were exposed to usual excitotoxic conditions, 100 μM CTZ for 5 min before incubation with 10 μM AMPA for 30 min, and toxicity assays or western blot (WB) analysis were carried out as described above to non-transfected oligodendrocytes experiments.

### Immunoprecipitation Assay

Oligodendrocyte cultures were treated in the absence (control) or presence of 100 μM CTZ and 10 μM AMPA for 30 min. Cells were washed three times in ice-cold PBS and lysed in RIPA buffer at different times (30, 60 or 90 min) after AMPA receptor activation. Lysates were pre-cleared with Sepharose 6B (Sigma-Aldrich) for 1 h at 4°C with a rotating wheel and then immunoprecipited overnight at 4°C with Protein A-Sepharose beads (Abcam) previously bound with rabbit anti-CK2α (Santa Cruz Technologies) or mouse anti-p53 (1C12; Cell Signaling) antibody. Beads were then washed three times with 1× lysis buffer, and immunoprecipitates were eluted with SDS gel loading buffer, boiled for 5 min and processed for immunoblotting for detection of p53 (using the mouse anti-p53) or phospho-p53 in Ser15 (using the rabbit anti-phospho-p53 (Ser15)), respectively. Total CK2α, p53 and β-actin were used as loading controls in whole cell lysate in the different situations. Immunoreactive bands were visualized using enhanced chemiluminescence detection kit, according to the manufacturer’s instructions (Supersignal; Thermo Scientific).

### Statistical Analysis

The analyzed number of samples was described in each experiment. All data are shown as mean ± SEM. Statistical analyses were performed using GraphPad Prism statistical software (version 5.0; GraphPad Software). Comparisons between multiple experimental groups were made using one-way ANOVA with Tukey’s *post hoc* test. For comparisons between a single experimental group and a control group, we used two-tailed Student’s *t*-test assuming equal variance. Statistical differences were considered significant where *p* < 0.05. All the images shown in this article represent the data obtained, at least, in three independent experiments with similar results.

## Results

### AMPA Receptor Stimulation Increases CK2 Activity in Oligodendrocytes

**Figure 1:**
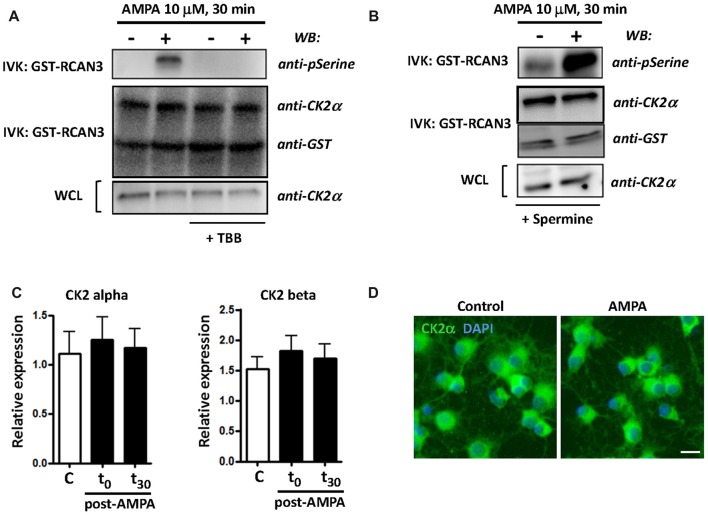
AMPA receptor stimulation increases casein kinase 2 (CK2) activity in oligodendrocytes. Cultured rat oligodendrocytes obtained from mixed glial cultures were pre-incubated with 5 μM TBB **(A)** or 10 μM spermine **(B)** and the excitotoxic insult (10 μM AMPA plus 100 μM CTZ) was performed for 30 min. In all cases, cells were lysed after 30 min post-stimulus. **(A,B)** CK2 was immunoprecipitated with 2 μg of anti-CK2α antibody and *in vitro* CK2 phosphorylation assay was performed using recombinant GST-RCAN3 in presence of 200 μM ATP (as described in “Materials and Methods” section). Immunocomplexes were subjected to *in vitro* kinase assay (IVK) followed by SDS-PAGE/immunoblotting (western blot, WB) and kinase activity were visualized using anti-phosphoserine antibody. In addition, the amount of CK2α and GST-RCAN3 present in each immunocomplex and the expression level of CK2α in whole cell lysates (WCL) were also determined by WB. Results are representative of three independent experiments. **(C)** mRNA for CK2α and CK2β subunits was analyzed by real-time qPCR, and relative expression of mRNA is shown. Analyses were performed in cultured oligodendrocytes treated with AMPA for 30 min and then mRNA was immediately extracted (t_0_) or 30 min later (t_30_). The data showed no significant modifications in CK2 expression levels at the time points evaluated. Values represent mean ± SEM (*n* = 3). **(D)** Immunofluorescence analysis of CK2α expression in cultured oligodendrocytes. Untreated control cells and treated for AMPA receptor activation were processed for CK2 immunofluorescence using the anti-CK2α antibody described before (green), and nuclei were stained with DAPI (blue). The staining was performed at 30 min post-stimulus and it could be observed that exposure to AMPA did not cause significant changes in CK2α protein expression levels, compared with control cells. Scale bar, 10 μm.

CK2 kinase activity is increased in some cellular stress conditions and this has deep consequences in cell viability (Sayed et al., 2001; Manni et al., 2012). To further define CK2 function in oligodendroglial death in AMPA receptor-induced excitotoxic events, we analyzed the activation of catalytic subunit CK2α and the expression of both catalytic and regulatory subunits of CK2 (CK2α and CK2β) under different experimental conditions. First, we carried out an *in vitro* kinase assay to examine the activity of CK2α (hereafter referred to as CK2) in oligodendrocytes after AMPA receptor stimulation (10 mM, 30 min) in the absence or in the presence of CK2 inhibitor TBB (5 mM; 3 h pre-treatment) or CK2 activator spermine (10 mM; 1 h pre-treatment). The kinase activity, determined by WB using an anti-phosphoserine antibody, showed that AMPA receptor stimulation induced potent increase of CK2 activity and this effect was not detected in the presence of TBB (Figure 1A). On the other hand, as expected, when oligodendrocytes were pretreated with CK2 activator spermine, AMPA-induced increase in CK2 activity was higher compared to that observed in cells treated only with agonist (Figure 1B). In addition, we also determined by WB the protein level of CK2α and GST-RCAN3 present in each immunocomplex as well as CK2α in whole cell lysates (WCL) what revealed similar amounts of these proteins in all the samples analyzed.

Additionally, and to determine whether AMPA-induced CK2 activity was correlated with modifications in the mRNA expression of catalytic and/or regulatory CK2 subunits we analyzed by RT-PCR their expression level in oligodendrocytes treated with AMPA (10 mM; 30 min) and evaluated immediately after treatment (*t* = 0) and 30 min later (*t* = 30). Consistent with immunoblot findings, we observed that CK2 subunits was constitutively expressed in oligodendrocytes but that their expression levels were not significantly increased after AMPA stimulation at the time points analyzed (Figure 1C). Similar results were obtained through immunofluorescence analysis on cultured oligodendrocytes, control and exposed to AMPA, using the anti-CK2α antibody (Figure 1D).

Taken together, these results indicate that AMPA excitotoxic insults in oligodendrocytes provoke a potent increase in CK2 kinase activity without causing significant changes in the CK2 mRNA or protein levels.

### CK2 Inhibition Rescues Cultured Oligodendrocytes From AMPA-Induced Excitotoxic Death

**Figure 2:**
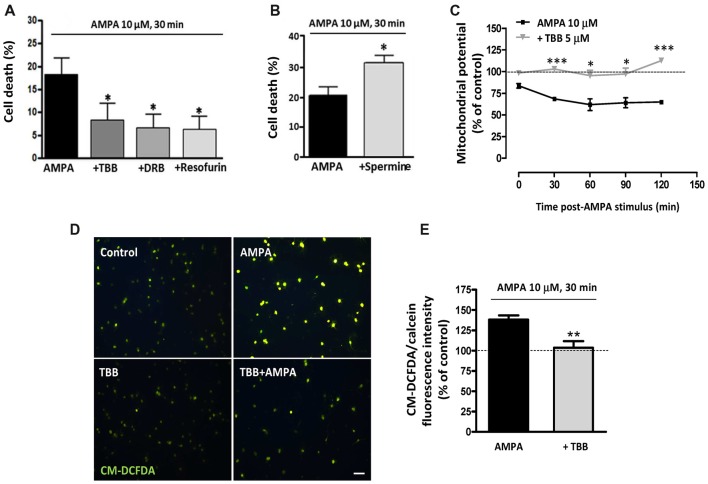
CK2 inhibition protects rat oligodendrocytes from AMPA excitotoxic insults. Oligodendrocyte cultures were isolated from mixed glial culture and used for toxicity assays after 2 days *in vitro* in differentiation SATO medium. **(A)** Activation of AMPA receptors with 10 μM AMPA applied together with 100 μM cyclothiazide for 30 min, caused oligodendroglial cell death as measured by calcein-AM assay 24 h later. AMPA-induced cytotoxicity was significantly reduced in presence of CK2 inhibitors, 5 μM TBB, 5 μM DRB or 5 μM resofurin and none of these CK2 inhibitors produced alterations in the oligodendrocyte viability when they were used without AMPA (data not shown). The data are shown as mean ± SEM (*n* ≥ 4; *p < 0.05 compared with cells treated with AMPA). **(B)** Pretreatment of cells with CK2 activator spermine (10 μM) increased cytotoxic consequences derived from AMPA receptor activation in oligodendrocytes. Bars represent mean ± SEM (*n* ≥ 3; *p < 0.05 compared with cells treated with AMPA). **(C)** CK2 inhibitor TBB attenuated loss of mitochondrial membrane potential induced by activation of AMPA receptors. Oligodendrocytes were exposed to 10 μM AMPA for 30 min in absence or presence of TBB (5 μM; 3 h pretreatment) and mitochondrial membrane potential was quantified by fluorimetry at the indicated times post-AMPA stimulus, after loading cells with dye JC-1 (3 μM, Molecular Probes). Decrease of mitochondrial potential triggered by excitotoxic insult was not observed in oligodendrocytes pretreated with 5 μM TBB. The data are represented as mean ± SEM compared with untreated control cells for each time point analyzed (*n* ≥ 4; *p < 0.05, ***p < 0.001). **(D,E)** ROS generation induced by activation of AMPA receptors was limited in the presence of CK2 inhibitor TBB. Oligodendrocytes were treated similarly as described above and ROS generation was determined immediately afterwards using the dye CM-DCFDA (10 μM, Molecular Probes). Cells were observed under fluorescence microscopy **(D)** and quantification of signal was measured using a Synergy-HT fluorimeter **(E)**. Histogram shows that levels of ROS induced by AMPA were attenuated in oligodendrocytes pretreated with TBB. Data represent mean ± SEM (*n* = 3; **p < 0.01, compared with cells treated only with agonist).

We have previously determined that rat oligodendrocytes are highly vulnerable to excitotoxic signals induced by overstimulation of AMPA receptors and that this toxicity is mediated by apoptotic mechanisms involving calcium overload, mitochondrial dysfunction, as well as activation of Bax, calpain and caspase -8, -9 and -3 (Sánchez-Gómez et al., 2003, 2011). Here, we investigated whether CK2, an enzyme closely linked with cell fate and whose activity we observed increased after AMPA treatment, contributes to oligodendroglial apoptotic program triggered by this glutamatergic agonist. To provide evidence that CK2 influences cellular viability, we induced AMPA receptor activation in oligodendrocytes in absence or presence of CK2 inhibitors and observed that cell death was significantly reduced when CK2 was inhibited (Figure 2A). So, activation of AMPA receptors with 10 mM AMPA, applied together with 100 mM CTZ for 30 min, caused oligodendroglial cell death (18.0 ± 3.9%), while oligodendrocytes treated previously with CK2 inhibitors (TBB, DRB or resofurin) were more resistant to these insults (7.9 ± 4.1%; 6.3 ± 3.4% and 5.7 ± 3.7%, respectively; Figure 2A). On the other hand, the presence of CK2 activator spermine increased almost twice AMPA-induced oligodendrocyte cell death (31.4 ± 2.6%), as shown in Figure 2B.

The above results provide evidence that CK2 is involved in excitotoxic insults. Since mitochondrial dysfunction is pivotal to this type of injury, we next analyzed whether CK2 also participate in the mitochondrial alterations that occur during oligodendrocyte excitotoxicity. Then, we determined mitochondrial depolarization and ROS production triggered by AMPA exposure (10 μM, in conjunction with 100 μM CTZ; 30 min) in absence or presence of CK2 inhibitor TBB (5 μM; 3 h pretreatment). We evaluated the mitochondrial membrane potential in these oligodendrocyte cultures, using the fluorescent probe JC-1, and performed its quantification at different times post-stimulus (Figure 2C). Consistent with the toxicity assays, we found that mitochondrial membrane depolarization resulting from those excitotoxic insults was attenuated in the presence of CK2 inhibitor TBB so that oligodendrocytes pretreated with TBB showed mitochondrial membrane integrity similar to the detected in untreated control cells, at all times analyzed (Figure 2C). Finally, cells subjected to excitotoxic stimulus were loaded with the probe CM-DCFDA for ROS detection and we observed that ROS generation triggered by AMPA receptor activation (up to 138.8 ± 4.4%) was significantly reduced in the presence of CK2 inhibitor TBB (103.7 ± 8.1%; Figures 2D,E).

Overall, these results indicate that pharmacological CK2 inhibition reduces both cell death as well as loss of mitochondrial membrane potential and acute generation of reactive oxygen species associated with AMPA-caused excitotoxicity in oligodendrocytes *in vitro*.

### Proapoptotic Contribution of CK2 Is Associated With MAPKs Activation Triggered by AMPA in Oligodendrocytes

**Figure 3:**
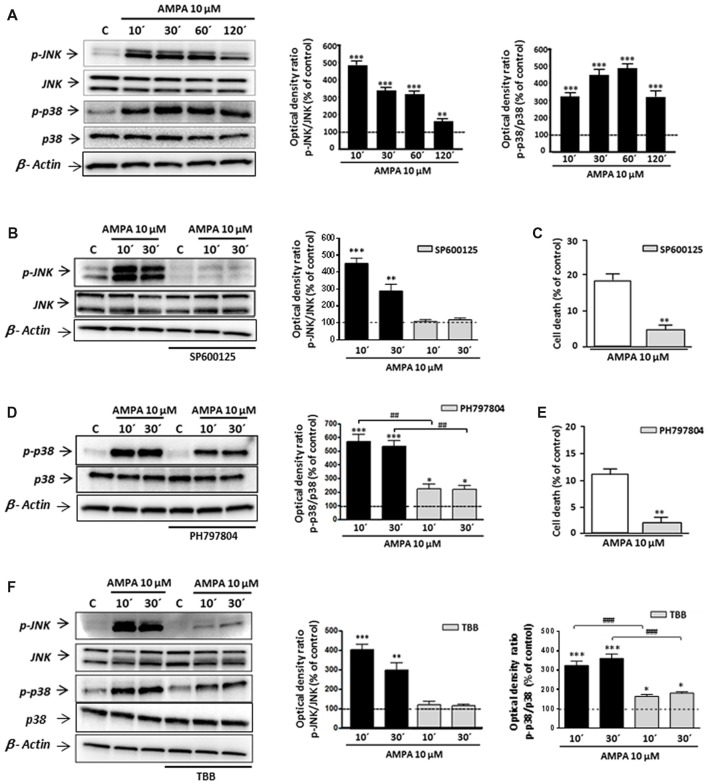
AMPA-induced c-Jun N-terminal kinase (JNK)/p38 apoptotic activation depends on CK2 activity. Oligodendrocytes were treated with 10 μM AMPA for 30 min, in the presence of 100 μM CTZ, and total lysates were harvested at the indicated times. When indicated, the stimulus was performed in the presence of the corresponding inhibitor. **(A)** Activation of AMPA receptors triggered an early and potent increase in the JNK and p38 phosphorylation levels at 10, 30, 60 and 120 min after AMPA treatment, determined by WB analysis. **(B,D)** The early AMPA-induced activation of JNK and p38 was prevented by the presence of 1 μM inhibitor SP600125 or PH797804, respectively. **(C,E)** The inhibition of JNK or p38 by SP600125 or PH797804, significantly reduced cell death induced by AMPA receptor activation in oligodendrocytes. **(F)** Intense JNK and p38 phosphorylation induced by exposure to AMPA was significantly reduced in the presence of CK2 inhibitor (TBB; 5 μM) at both times analyzed (10 and 30 min post-stimuli). *p < 0.05, **p < 0.01, ***p < 0.001 (cells treated with AMPA vs. control cells); ^##^*p* < 0.01, ^###^*p* < 0.001 (cells treated with each mitogen-activated protein kinases (MAPKs) inhibitor vs. cells treated with only AMPA).

Although some studies have described that CK2 may protect against cell death in different contexts (Ahmad et al., 2008; Turowec et al., 2010) other works indicated that, under certain cell-specific conditions, CK2 can act as pro-death molecule, for instance, via CK2-JNK interaction (Min et al., 2003; Hilgard et al., 2004; Ka et al., 2015). With this premise, we tried to define the mechanism by which CK2 inhibition rescues oligodendrocytes from excitotoxic death and, specifically, if mitogen-activated protein kinase (MAPK)s activated by stress JNK and p38 are part of this pathway. For that, we first examined, by WB with specific antibodies, the phosphorylation (activation) state of JNK and p38 in total cell lysates obtained from control and AMPA-exposed oligodendrocytes. As depicted in Figure 3, phosphorylation levels of both enzymes were early and robustly induced after agonist exposure, reaching values for phospho-JNK of 505.3 ± 28.2%; 352.4 ± 20.6%; 332.0 ± 17.4% and 167.0 ± 17.3% at 10, 30, 60 and 120 min post-stimulus, respectively, and for phospho-p38 of 314.9 ± 16.4%; 438.3 ± 25.5%; 471.7 ± 22.7% and 310.4 ± 27.3% at the same times analyzed, always compared to untreated control cells (100%; Figure 3A). The presence of inhibitor of JNK SP600125 significantly reduced AMPA-induced JNK activation (108.1 ± 11.9% and 118.2 ± 10.5% compared with 451.0 ± 31.4% and 288.3 ± 39.36% observed in AMPA-treated cells without inhibitor, at 10 and 30 min after stimulus, respectively; Figure 3B). Similarly, the activation/phosphorylation of p38 triggered by AMPA was significantly reduced when its inhibitor PH797804 was present during the treatment (Figure 3D). This reduction of phosphorylation levels, both JNK and p38, supported the effectiveness of these inhibitors in the conditions used, so we used them in the following assays.

It should be mentioned that the potent and fast induction of JNK and p38 activation is consistent with their role as members of death signaling axis, triggered by toxic signals and mediating in the activation of downstream apoptotic molecules. In order to check the involvement of JNK and/or p38 in the apoptotic process triggered by AMPA in oligodendrocytes, we developed toxicity assays in the presence of their specific inhibitors, SP600125 and PH797804, respectively. As we showed in Figures 3C,E the oligodendroglial death caused by AMPA exposure (10 μM; 30 min) was significantly reduced in the presence of each inhibitor. So, the values of cell death were of 5.3 ± 1.2% in presence of SP600125, compared with 18.2 ± 2.0% for cells only treated with AMPA (Figure 3C) and 2.6 ± 2.2% in presence of PH797804, compared to 10.8 ± 2.4% in absence of this inhibitor (Figure 3E).

Then, and to determine the possible relationship between CK2 and JNK/p38 axis after AMPA receptor stimulation in oligodendrocytes, we determined the phosphorylation state of both kinases in AMPA treated cells in absence or presence of CK2 inhibitor TBB (Figure 3F). We found that AMPA-induced phosphorylation of JNK (403.2 ± 29.7%; 299.3 ± 38.7% at 10 and 30 min after stimulus, respectively) and p38 (324.3 ± 23.1%; 361.0 ± 22.6% at the same time post-stimulus), was significantly reduced in TBB pre-treated oligodendrocytes (122.5 ± 16.5%; 116.7 ± 8.5% for phospho-JNK and 164.7 ± 10.5%; 181.7 ± 7.7% for phospho-p38 at the times indicated, respectively).

These results indicate that TBB, inhibitor of CK2, regulates the MAPK signaling pathway induced by AMPA in oligodendrocytes, so that the level of phosphorylation of JNK and p38 depends on the degree of activation of CK2. This molecular interaction could have important implications in the resistance/vulnerability of the oligodendrocytes subjected to excitotoxic conditions.

### CK2-JNK Are Involved in AMPA-Caused Damage Oligodendrocyte in Isolated Optic Nerves

**Figure 4:**
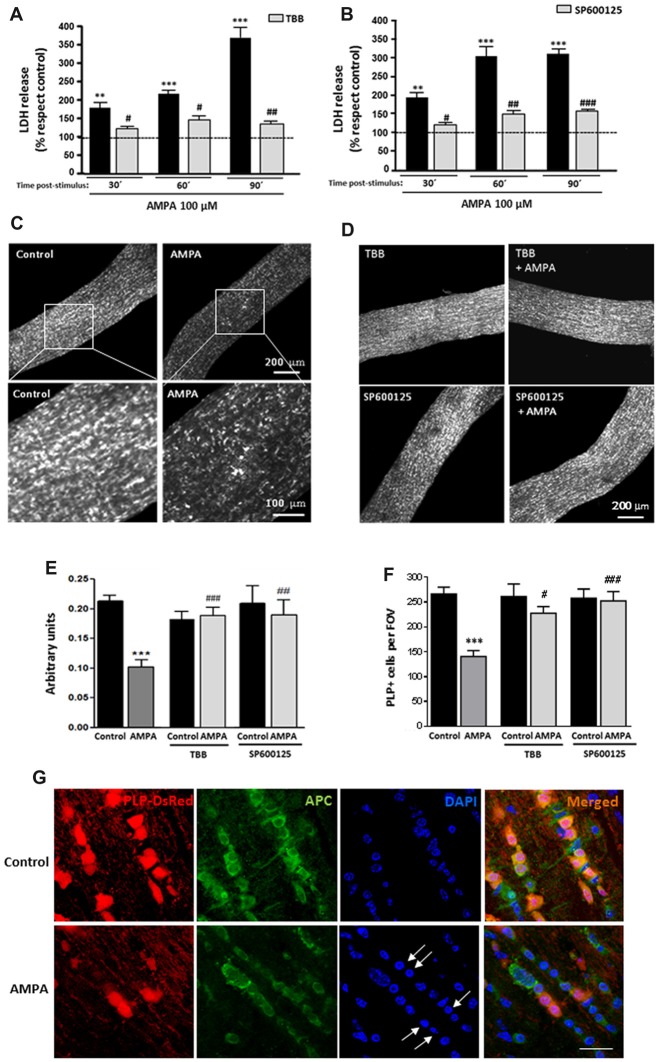
AMPA provokes oligodendroglial damage in isolated optic nerves from PLP-DsRed transgenic mice. Isolated optic nerves from PLP-DsRed transgenic mice were preincubated in artificial CSF medium in absence or presence of TBB (25 μM; 3 h) or SP600125 (20 μM; 1 h) and subjected to excitotoxic insult by stimulation of AMPA receptors (100 μM CTZ plus 100 μM AMPA) during 2 h. **(A,B)** Lactate dehydrogenase (LDH) release quantification at 30, 60 and 90 min post-stimulus showed that TBB or SP600125 pre-treated optic nerves exhibited a significant reduction in LDH release at all post-stimulus times analyzed. **p < 0.005; ***p < 0.0001 (optic nerves treated with AMPA* vs* optic nerves control); ^#^*p* < 0.05; ^##^*p* < 0.01; ^###^*p* < 0.001 (vs. optic nerves treated only with AMPA). **(C,D)** Representative fields of z-stacks of optic nerves from PLP-DsRed transgenic mice control or stimulated with AMPA alone **(C)** or in presence of inhibitors TBB or SP600125 **(D)**. Note a loss of oligodendrocytes in optic nerves exposed to AMPA, which was prevented by incubation of agonist in presence of TBB or SP600125. **(E)** Quantification of the fluorescence signal emitted by oligodendrocytes of optic nerves from P25 PLP-DsRed mice, expressed as arbitrary units of fluorescence. ***p < 0.001 (optic nerves treated with AMPA vs. optic nerves control); ^##^*p* < 0.01, ^###^*p* < 0.001 (vs. optic nerves treated only with AMPA). **(F)** Cell counts of PLP+ oligodendrocytes were performed and data were represented as mean number of cells (±SEM, *n* > 4 animals) in a constant volume (FOV), as detailed in “Materials and Methods” section. ***p < 0.001 (optic nerves treated with AMPA vs. optic nerves control); ^#^*p* < 0.05, ^###^*p* < 0.001 (vs. optic nerves treated only with AMPA). Both quantifications show that oligodendrocyte loss induced by AMPA excitotoxic insult was inhibited by the presence of TBB or SP600125 inhibitors. **(G)** Optic nerves from PLP-DsRed transgenic mice were processed for immunofluorescence using antibodies to APC and nuclei were stained with DAPI. Treatment with AMPA induced a dramatic fluorescence loss in both PLP and APC oligodendroglial markers, indicating severe oligodendrocyte damage. In addition, DAPI labeling revealed nuclei condenzation in AMPA-treated optic nerves (arrows). Scale bar 20 μm.

To further investigate the deleterious effects of AMPA in a more integral preparation, we analyzed the AMPA receptor-mediated injury to intact optic nerves, a preparation which we have previously used to test receptor-mediated toxicity (Sánchez-Gómez et al., 2003; Domercq et al., 2010; Mato et al., 2013; González-Fernández et al., 2014). In this case, we used isolated intact optic nerves from transgenic mice (PLP-DsRed) which allows identification of the soma and the processes of oligodendrocytes by red fluorescence. Freshly isolated optic nerves from P25 mice were exposed for 2 h to AMPA plus CTZ (100 μM each) in oxygen-saturated artificial CSF, in presence or absence of inhibitors TBB or SP600125 and cell damage was monitored by measuring LDH release at 30, 60 and 90 min after the withdrawal of the stimuli (Figures 4A,B). Incubation of optic nerves with the agonist induced a marked damage that was increasing over time after AMPA exposure, reaching values for LDH release 90 min later, up to near four times higher than the control (369.1 ± 35.2% respect to 100% control). This harmful effect was absent when nerves were preincubated with CK2 inhibitor (TBB; 25 μM, 3 h; Figure 4A) or with JNK inhibitor (SP600125; 20 μM, 1 h; Figure 4B) and maintained 2 h with AMPA stimulus in the incubation medium. The protective effect of both inhibitors was statistically significant at all times post-stimulus analyzed, included at 90 min after removal of the agonist (Figures 4A,B).

Additionally and when LDH release assays were completed, optic nerves were fixed and high-resolution confocal z-sections of whole-mounted nerves were obtained. As showed in representative images of optic nerves, the incubation of isolated intact optic nerves with AMPA induced a potent loss of oligodendrocytes and their processes (Figure 4C), which was prevented in the presence of TBB or SP600125 (Figure 4D). The quantification of fluorescent signal in the z-stacks of whole nerves corroborated this effect, and showed that the disappearance of the half of DsRed fluorescent caused by AMPA (arbitrary units 0.10 ± 0.01 respect to 0.21 ± 0.01 of control) was abolished when the inhibitors were added (arbitrary units 0.19 ± 0.02 for TBB and 0.19 ± 0.04 for SP600125; Figure 4E). In addition, we counted individually the PLP-DsRed^+^ oligodendrocytes in these preparations and we observed that AMPA treatment also reduced by almost half the cellular number compared with control (140.5 ± 13.9 vs. 266.2 ± 13.9 per FOV, respectively; Figure 4F). As in the previous analysis, the inhibition of CK2 or JNK during the AMPA receptor activation completely avoided the effect caused by agonist (227.5 ± 13.6 cells for TBB and 252.5 ± 18.9 cells for SP600125).

Finally and to corroborate the effect of AMPA exposure in viability of oligodendrocytes we assayed the status of the oligodendrocyte marker APC (Ab-7) and their nuclei by DAPI staining, in cryosections of optic nerves from control and AMPA-treated PLP-DsRed mice (Figure 4G). Then, immunohistochemistry of optic nerves confirmed that oligodendrocytes were severely damaged by AMPA as they showed loss of PLP-DsRed, as it had previously been described, and reduction in the APC expression and these alterations were accompained with chromatin condenzation revealed by DAPI staining (arrows in Figure 4G).

These results in whole optic nerves *ex vivo* further assessed the observations described above showing that activation of AMPA receptors triggered apoptotic cell death in oligodendrocytes and the tandem CK2-JNK plays a key role in execution of this apoptotic program. These data, in combination with results obtained from *in vitro* kinase assay, suggests that a greater degree of vulnerability of oligodendrocytes to AMPA may be linked to a higher degree of activation of CK2 kinase, which in turn, mediates subsequent JNK activation that culminates in cell death.

### Gene Silencing of CK2 Reduced AMPA-Induced Oligodendrocyte Cell Death and Associated JNK Activation

**Figure 5:**
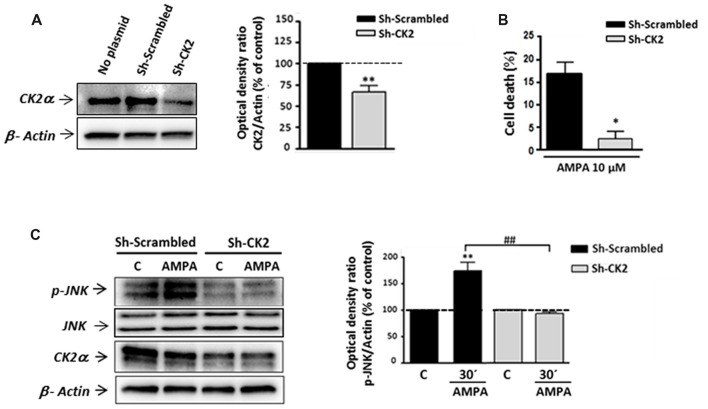
Gene silencing of CK2 mimics its pharmacological inhibition with pro-survival effects on oligodendrocytes exposed to AMPA. Isolated oligodendrocytes from mixed glial cultures were immediately transfected with Sh-Scrambled or Sh-CK2 (as described in “Materials and Methods” section) and then were maintained in culture until used. **(A)** WB analysis of endogenous CK2α expression in lysates of non-transfected cells (no plasmid) and cells transfected with shRNAs directed at CK2 (Sh-CK2) or the control sequence (Sh-Scrambled). Sh-CK2 reduced the expression of CK2 compared with non-transfected cells or transfected with control shRNA. The analysis of the β-actin was performed as an internal load control. **p < 0.01 (vs. Sh-Scrambled transfected cells). **(B)** Transfected oligodendrocytes were exposed to usual excitotoxic conditions (10 μM CTZ for 30 min), and cell viability was assayed 24 h later using calcein-AM. Knocking down-CK2 cells are less vulnerable to AMPA-induced cell death than Sh-Scrambled transfected cells. *p < 0.05 (vs. Sh-Scrambled transfected cells treated with agonist). **(C)** WB analysis of JNK phosphorylation state in transfected oligodendrocytes after excitotoxic insults. Cells were transfected with Sh-Scrambled or Sh-CK2, maintained in control situation or treated with AMPA (10 μM, 30 min) and subjected to western blotting with specific antibodies against phospho-JNK, total JNK, CK2α and β-actin, 30 min post-stimulus. Quantification shows that JNK phosphorylation by AMPA exposure was significantly blocked in Sh-CK2 transfected cells as compared to Sh-Scrambled transfected cells, without changing expression levels of total JNK. Detection of CK2α in these samples corroborated that expression of kinase was notably reduced in Sh-CK2 transfected oligodendrocytes. Analysis of β-actin was performed as an internal load control. **p < 0.01 (vs. control situation without AMPA treatment); ^##^*p* < 0.01 (vs. Sh-Scrambled transfected cells treated with agonist).

To confirm the contribution of CK2-JNK to excitotoxic cascade initiated by AMPA in oligodendrocytes, we next knocked down endogenous CK2 expression in these cells by transfecting CK2 shRNA. First, we observed that CK2 shRNA (sh-CK2) but not control shRNA (sh-Scrambled) significantly reduced the endogenous level of CK2 up to 73.28 ± 2.65% (Figure 5A). Next, oligodendrocytes from each of these experimental conditions were subjected to AMPA treatment and then we quantified the toxicity level caused by the agonist in these cells (Figure 5B). Consistent with the cytoprotective effect achieved by CK2 pharmacological inhibitors (TBB, DRB or resofurin), we observed that AMPA-triggered oligodendrocyte death was significantly reduced in cells transfected with sh-CK2, compared with sh-Scrambled transfected cells (2.3 ± 1.8% vs. 16.6 ± 2.8%, respectively). These results confirm that CK2 is involved in the harmful process initiated by AMPA in oligodendrocytes.

Because CK2 and JNK operate together in triggering cell death events, and based on our results described above, we tried to determine if knocking down of endogenous CK2 affects JNK phosphorylation after AMPA exposure. As showed in Figure 5C, we observed significant increase of JNK phosphorylation level in Sh-Scrambled transfected cells after AMPA receptors stimulation (173.6 ± 15.8%, respect to untreated cells, 100%). However, knocking down CK2 oligodendrocytes subjected to the same AMPA stimulus showed no increment in JNK phosphorylation degree, with values close to the control (92.4 ± 3.8% respect to 100% control). It should be noted that in these knocking down cells could be verified a remarkable decrease in the expression of CK2, both in control cells and treated with AMPA (Figure 5C).

Overall, these observations are consistent with pharmacological CK2 inhibition and further assess that CK2 plays a key role in AMPA-induced oligodendroglial toxicity by interfering with JNK pro-apoptotic activation.

### p53 Is a Downstream Pro-apoptotic Effector of CK2/JNK Signaling Cascade in AMPA-Induced Oligodendrocyte Cell Death

One potential target of pro-apoptotic signaling by JNK is the tumor suppressor p53 (Dhanasekaran and Reddy, 2008; Choi et al., 2011; Saha et al., 2012). Additionally, the involvement of CK2 kinase in the p53 phosphorylation process is essential for activation of p53 and its entry into cell pro-death programs and apoptosis (Sayed et al., 2001; Meek and Cox, 2011; Rao et al., 2014). Moreover, p53 participates in neuronal glutamatergic excitotoxicity (Choi et al., 2011; Shah et al., 2014), so we explored the role of p53 in oligodendroglial excitotoxicity and its potential interaction with CK2-JNK.

**Figure 6:**
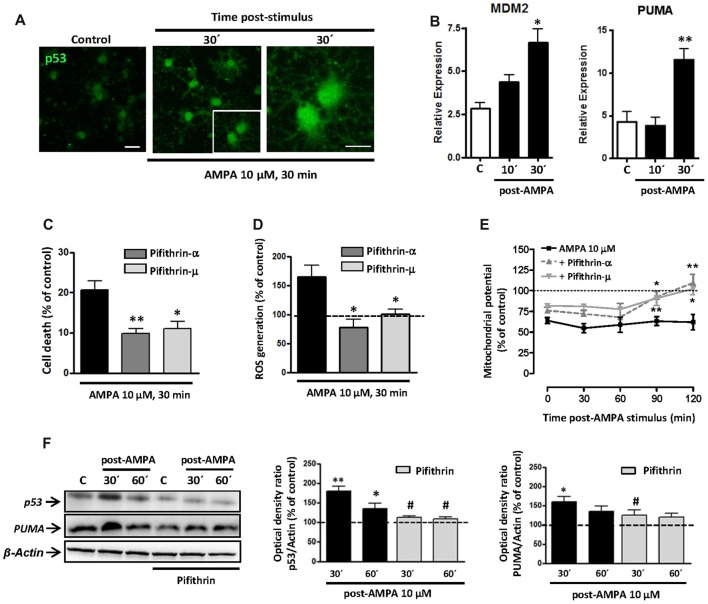
AMPA excitotoxic insult induces p53 stabilization and transcriptional activation in oligodendrocytes, which is inhibited by pifithrin. Oligodendrocytes were exposed to 10 μM AMPA together with 100 μM CTZ for 30 min. **(A)** Immunofluorescence analysis of p53 expression. After AMPA receptor activation, at 30 min post-stimulus, cells were processed for p53 immunofluorescence using the p53 (FL-393) antibody, which recognized total p53. Exposure to AMPA triggered a notable increase in the expression of total p53 (green). Scale bar, 20 μm (left and central images); 10 μm (right image). **(B)** RT-PCR analysis for expression of p53 transcriptional targets, MDM2 and PUMA, showed a significant rise in their relative expression in cultured oligodendrocytes after AMPA receptor activation. (*n* ≥ 3; *p < 0.05, **p < 0.01 respect to untreated control cells). **(C)** AMPA-induced excitotoxicity was significantly reduced in the presence of p53 inhibitors, pifithrin-β (20 μM) or pifithrin-μ (1 μM), preincubated for 1 h before the activation of AMPA receptors. (*p < 0.05, **p < 0.01, respect to cells treated only with AMPA). **(D)** ROS generation triggered by activation of AMPA receptors was attenuated by p53 inhibitors. Oligodendrocytes were exposed to 10 μM AMPA for 30 min in absence or presence of pifithrin and ROS level was immediately determined using the dye CM-DCFDA (10 μM, Molecular Probes) and measuring the signal in a Synergy-HT fluorimeter. Results showed that levels of ROS induced by AMPA were attenuated in oligodendrocytes pretreated with both pifithrin-α or pifithrin-μ. Data represent mean ± SEM (*n* = 3; *p < 0.05, compared with cells treated only with agonist). **(E)** In addition, mitochondrial membrane potential was quantified by fluorimetry at different times after AMPA stimulus, by loading cells with dye JC-1 (3 μM, Molecular Probes). Mitochondrial depolarization induced by excitotoxic insult was attenuated in pifithrin-α and pifithrin-μ pre-treated oligodendrocytes, as compared with cells treated only with agonist. Data are represented as mean ± SEM compared with cells treated only with AMPA for each time point analyzed (*n* ≥ 3; *p < 0.05; **p < 0.01). **(F)** Oligodendrocytes were preincubated with pifithrin-μ (1 μM) before AMPA receptor activation and total protein was collected 30 and 60 min later for WB analysis. The presence of p53 inhibitor pifithrin-μ during excitotoxic signals reduced p53 and PUMA accumulation induced by AMPA. The analysis of β-actin was performed as an internal load control and was used to quantify the expression level of p53 and PUMA in each condition. (*p < 0.05, **p < 0.01 respect to control cells; ^#^*p* < 0.05 respect to cells treated only with AMPA).

Initially, we analyzed the status of p53 in oligodendrocytes and, as shown in Figure 6A, we detected an increase in the levels of total p53 in oligodendrocytes following the stimulation of AMPA receptors (10 μM, 30 min), analyzed at 30 min after the withdrawal of the agonist. This change can indicate an increase in the stability of p53, which can be associated with its role as transcription factor (Allende-Vega et al., 2013; Loughery and Meek, 2013) and its consequent participation in apoptosis.

To analyze the possible transcriptional activity of p53 in oligodendrocyte excitotoxicity, we determined by qPCR the gene expression of molecules interacting with the p53 system, as MDM2 and PUMA. As shown in Figure 6B, activation of AMPA receptors in oligodendrocytes caused a rapid and sustained increase in mRNA levels of MDM2, which was statistically significant at 30 min after application of the agonist. On the other hand, activation of receptors in oligodendrocytes caused a significant increase in PUMA levels (Figure 6B), a small protein of Bcl-2 family that is induced by transcriptional activation of p53 and acts as a key mediator of cytosolic proapoptotic p53 function. These data are in agreement with those documented by other authors in p53-dependent apoptotic contexts (Yu et al., 2003; Antony et al., 2012).

Based on these results, we examined the contribution of p53 on excitotoxic damage by evaluating AMPA-induced cell death in presence of p53 inhibitors, pifithrin-α and pifithrin-μ. Pre-treated oligodendrocytes with 20 μM pifithrin-α or 1 μM pifithrin-μ were less vulnerable to AMPA exposure, so the cell death was of 9.8 ± 1.2% or 11.0 ± 1.8%, respectively, compared to 20.7 ± 2.1% observed in cells treated only with agonist (Figure 6C). Furthermore, p53 inhibition also reduced AMPA-triggered mitochondrial dysfunction analyzed by ROS production and loss of mitochondrial transmembrane potential (Figures 6D,E). Thus, we observed ROS levels significantly increased after activation of AMPA receptors (up to 164.2 ± 20.8%), an effect that was abolished in the presence of pifithrin-α or pifithrin-μ (77.2 ± 14.5% or 100.4 ± 7.8%, respectively; Figure 6D). Besides, JC-1 probe fluorescence quantification at different times post-stimulus showed that AMPA receptor activation induced a loss of mitochondrial potential post-stimulation reaching minimum levels between 30 and 60 min (58.6 ± 5.6%; 59.2 ± 9.3%, respectively). In contrast, the mitochondrial potential in p53 inhibitor pre-treated oligodendrocytes showed a significant recovery, with values near to control after 90 min post-stimulus (Figure 6E).

Finally, we analyzed by WB the effect of pifithrin on total p53 and PUMA protein expression following the excitotoxic insult (Figure 6F). AMPA treatment provoked significant increases in p53 and PUMA protein level (180.3 ± 11.5% and 159.7 ± 13.5%, respect to 100% control), observed at 30 min post-stimulus, which were attenuated by pifithrin in both cases (111.5 ± 4.4% for p53 and 124.7 ± 14.2% for PUMA).

**Figure 7:**
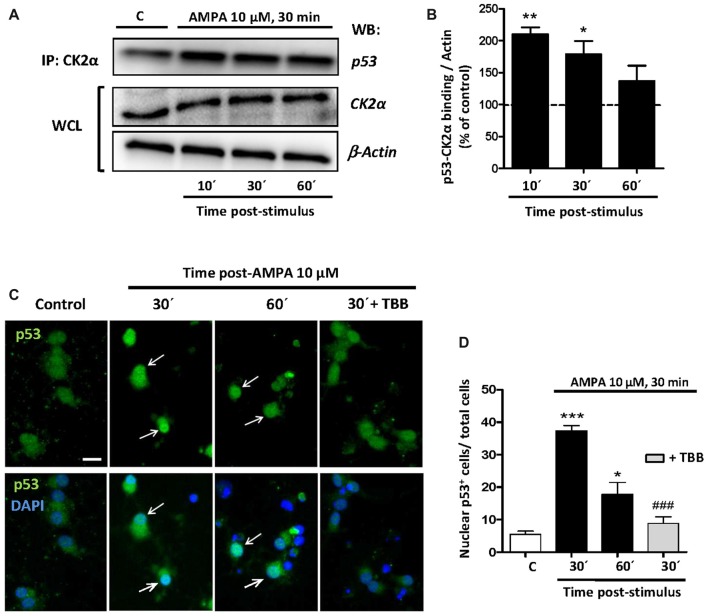
CK2 directly interacts with p53 under conditions of AMPA-induced excitotoxicity in oligodendrocytes. **(A)** Oligodendrocytes in culture were subjected to excitotoxic insult and cell lysates were obtained at 10, 30 and 60 min after AMPA receptor activation. Co-immunoprecipitation assays were performed in the presence of rabbit polyclonal anti-CK2α antibody (IP: CK2α) and the immunoprecipitates were analyzed by WB using a mouse anti-p53 antibody. CK2α and β-actin were measured as internal loading controls in WCL. The image corresponds to one representative co-immunoprecipitation assay where it is observed that activation of AMPA receptors in oligodendrocytes provokes a direct interaction between CK2 and p53. **(B)** Quantification of p53-CK2α binding after co-immunoprecipitation assay in the experimental conditions indicated (*n* = 3; *p < 0.05; **p < 0.01 respect to control). **(C)** Immunofluorescence analysis of total p53 expression after AMPA receptors activation in absence or presence of CK2 inhibitor TBB. After AMPA treatment, oligodendrocytes were fixed, processed for immunofluorescence with an anti- total p53 antibody (p53 FL-393; green) and nuclei were stained with DAPI (blue). Images show that the increase in p53 activation and its translocation to the nuclei, examples indicated by arrows, was reduced by the CK2 inhibitor TBB. Scale bar, 10 μm. **(D)** Quantification of the number of p53 nuclear events determined by counting the cells with p53 in DAPI-positive region as fraction of total cells present in each condition. Cell counts were performed on a minimum of 10 independent fields (30 fields/3 coverslips/treatment) of images captured with a 20× objective, and all experimental conditions were always paired with corresponding internal controls (*p < 0.05; ***p < 0.001 respect to control; ^###^*p* < 0.001 respect to AMPA after 30 min).

Once the participation of p53 in the excitotoxic process was established, we analyzed the possible linkage between p53, CK2 and JNK based on that CK2 can directly phosphorylate p53 and modulate its transcriptional activity (Sayed et al., 2001; Keller and Lu, 2002), or indirectly through the CK2-initiated pathway and executed by JNK. To study the possibilities we first performed co-immunoprecipitation assays between CK2-p53 in which we immunoprecipitated the CK2α protein in control oligodendrocytes and treated with 10 μM AMPA for 30 min and recollected at 10, 30 and 60 min later. Then, we detected by WB the presence of p53 in the immunoprecipitaded samples (Figure 7A).

The results showed that the amount of p53 linked to CK2α rapidly increased after AMPA treatment, indicating a fast, powerful and direct molecular interaction between CK2α and p53. However, this union decreased over time so that at 10 min after AMPA elimination the binding p53-CK2α reached a level of 210.0 ± 19.1%, at 30 min was of 179.0 ± 20.2%, and at 60 min was of 137.1 ± 23.9%, compared with 100% of control (Figure 7B).

In addition, by immunofluorescence staining we observed that AMPA early induced both p53 activation and its nuclear localization and these events were significantly reduced in oligodendrocytes pre-treated with CK2 inhibitor TBB (5 μM; pretreatment 3 h; Figure 7C). To quantify this effect, we counted the oligodendrocytes that showed p53 in the nucleus (stained with DAPI) in cultures exposed to different situations and we could observed that AMPA caused a significant increase in nuclear location of p53 after 30 min (37.3 ± 1.6 cells per field) and 60 min (17.8 ± 3.6), compared to control cells (5, 4 ± 0.9 cells per field). However, the presence of TBB significantly reduced the number of oligodendrocytes showing nuclear p53 after AMPA exposure and analyzed 30 min later (8.8 ± 1.9 cells per field; Figure 7D). Together, these data suggest that CK2 participates, at least in part, in p53 activation in oligodendrocytes subjected to the stimulation of AMPA receptors.

### Ser15 Phosphorylation of p53 Following AMPA Exposure Is JNK and CK2 Dependent

Apoptotic stimuli can lead to rapid and substantial multisite phosphorylation of p53 by different kinases, nucleated initially through phosphorylation of Serine 15 (Jones et al., 2005; Meek, 2009). This suggests a key and possibly universal role in promoting p53-transcription function (Loughery et al., 2014). In addition, this post-transcriptional modification of p53 has been related with its mitochondrial insertion and pro-apoptotic interaction with proteins of Bcl-2 family (Park et al., 2005; Nieminen et al., 2013; Wang et al., 2015).

**Figure 8:**
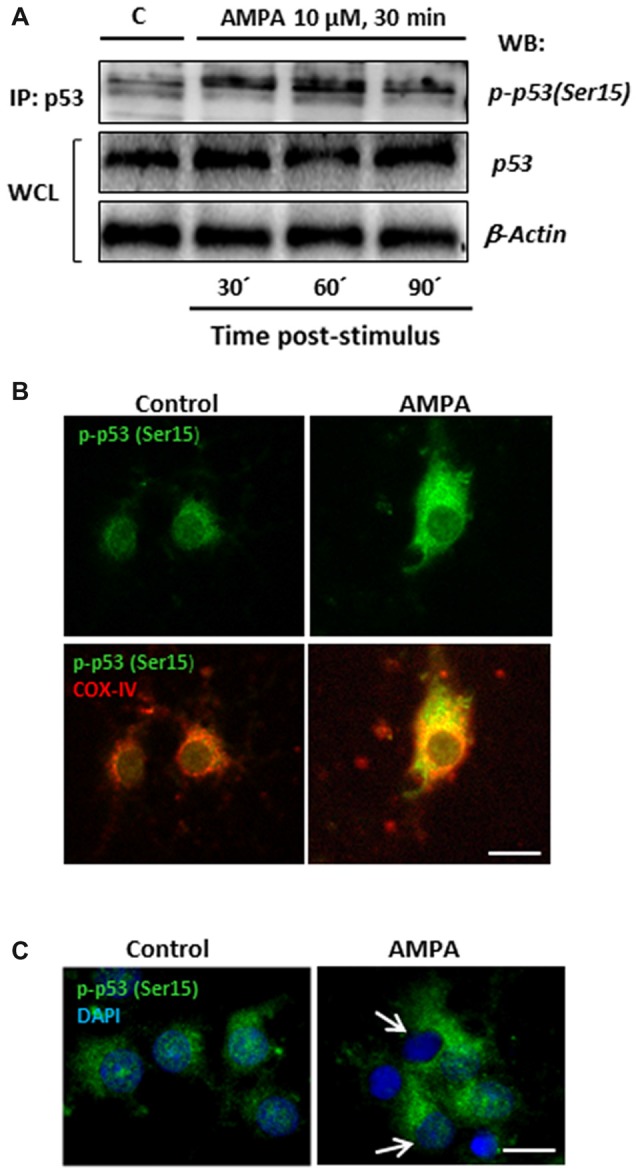
AMPA leads to sustained p53 activation through its phosphorylation in Ser15 and its mitochondrial accumulation. **(A)** Immunoprecipitation assays were performed in cultured oligodendrocytes after AMPA receptor activation with 10 μM AMPA plus100 μM CTZ for 30 min. Cell lysates from oligodendrocytes were harvested at 30, 60 and 90 min post-stimulus and immunoprecipitated with a mouse anti-p53 antibody (total p53; IP:p53). Immunoprecipitates were analyzed by WB using the rabbit antibody against phospho-p53 (Ser15). Total p53 and β-actin in WCL were used as internal loading controls. The image corresponds to one representative immunoprecipitation assay where it is observed an increase in the p53 phosphorylation in Ser15 in response to excitotoxic insults in cultured oligodendrocytes. **(B)** AMPA induced mitochondrial accumulation of phospho-p53 (Ser15). Oligodendrocytes were treated for 30 min with 10 μM AMPA plus 100 μM CTZ and fixed and processed for immunofluorescence using the specific antibody phospho-p53 (Ser15), which only recognizes p53 when it is phosphorylated at this residue (green), and COX-IV antibody to detect mitochondria (red). Exposure to AMPA triggered p-p53 (Ser15) increase with cellular localization coincident with the mitochondrial environment (yellow). Scale bar, 10 μm. **(C)** Immunofluorescence images of oligodendrocytes control and exposed to AMPA where it is remarkable the accumulation of p-p53 (Ser15) induced by AMPA as well as the total absence of p-p53 (Ser15) in the nuclei, contrasted with DAPI (arrows). Scale bar: 10 μm.

With the aim of investigating this possibility in our experimental paradigm, we carried out immunoprecipitation assays to detect the presence of p53 phosphorylated in the residue Serine15, p-p53 (Ser15), in control oligodendrocytes and subjected to excitotoxic damage (10 μM AMPA in presence of 100 μM CTZ for 30 min). For that, total p53 protein was immunoprecipitated by binding agarose beads to an anti-total p53 antibody, applying the protocol described in “Materials and Methods” section (IP: p53; Figure 8A). Then, the immunoprecipitates were subjected to WB using a specific antibody (anti-p-p53 (Ser15)) to detect the presence of the Ser15 phosphorylated p53 fraction both in control and treated oligodendrocytes. This specific antibody detects endogenous levels of p53 only when phosphorylated at Ser15 and does not cross-react with p53 phosphorylated at other sites.

As shown in Figure 8A, there was an increase in p53 phosphorylation at Ser15 induced by AMPA, especially remarkable at 30 and 60 min after withdrawal of the AMPA stimulus. In addition, the presence of p-p53 (Ser15) was also determined by immunocytochemistry in oligodendrocytes control and stimulated with 10 μM AMPA for 30 min (Figure 8B). The treatment induced a notable increase in the presence of p-p53 (Ser15) and, importantly, this phosphorylated p53 was predominantly located in cell regions stained with COX-IV antibody, suggesting a mitochondrial redistribution triggered by AMPA receptor activation. In addition, although some studies have also described the presence of p-p53 (Ser15) at the nuclear level (Li et al., 2010; Jin et al., 2013), we have seen total absence of this residue in the nucleus of oligodendrocytes after AMPA receptor stimulation (Figure 8C).

Finally, and based on the literature describing that JNK and p53 may also directly interact (Saha et al., 2012; Shi et al., 2014), we next analyzed the behavior of p53 and p-p53 (Ser15) in oligodendrocytes stimulated with AMPA in absence or presence of JNK inhibitor SP600125.

**Figure 9:**
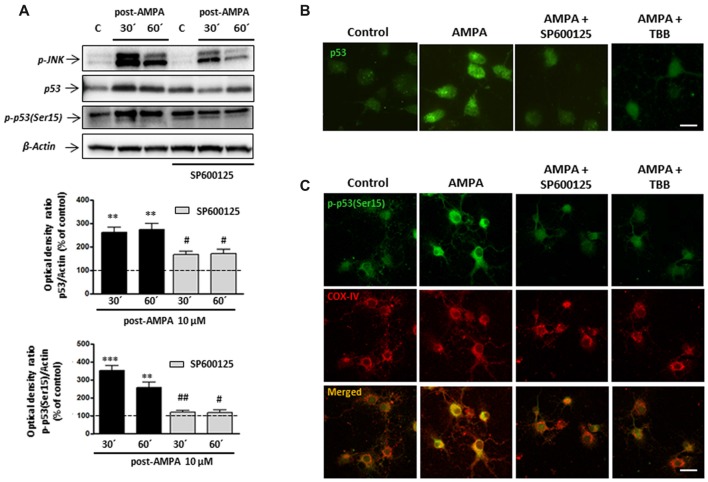
Phosphorylation of p53 at Ser15 induced by AMPA is JNK/CK2 dependent. Oligodendrocytes were treated for 30 min with 10 μM AMPA plus 100 μM CTZ, in absence or presence of JNK or CK2 inhibitors, and the different assays were carried out at the indicated times. **(A)** WB analysis of total p53 and p-p53(Ser15) at 30 and 60 min after excitotoxic stimulus, in absence or presence of 1 μM SP600125. Exposure to AMPA induced an increase in expression levels of both total p53 and p-p53(Ser15), which were abolished by the JNK-inhibitor SP600125. The level of JNK phosphorylation was used as a control of the effectiveness of the treatment and β-actin was the internal loading control. **p < 0.01, ***p < 0.001 (cells treated with AMPA respect to control cells); ^#^*p* < 0.05, ^##^*p* < 0.01 (cells treated with SP600125 respect to cells treated with only AMPA, at the same time). **(B)** AMPA triggered increases in p53 expression level, revealed with an antibody against total p53, which was reverted by JNK inhibitor SP600125 and CK2 inhibitor TBB. Scale bar: 10 μm. **(C)** Cells were treated with AMPA, as indicated above, in the presence of SP600125 or TBB and fixed at 30 min after stimulus. Immunofluorescence analysis using the specific antibody against p-p53(Ser15; green) showed that AMPA-induced activation of p53 through its phosphorylation in Ser15 was not detected in presence of JNK or CK2 inhibitors and neither its mitochondrial accumulation (revealed by COX-IV antibody; red). Scale bar: 10 μm.

As shown in Figure 9A, WB analysis of p53 levels after AMPA receptor stimulation (AMPA 10 μM plus CTZ 100 μM, 30 min), showed that excitotoxic insults increased p53 expression at 30 and 60 min after stimulus (260.7 ± 22.5% and 273.3 ± 26.0%, respectively and compared to control cells). The presence of JNK inhibitor SP600125 1 μM significantly reduced that effect (166.7 ± 14.5% y 169.3 ± 17.8%, respectively, compared to control cells). In addition, we detected a robust AMPA-triggered phosphorylation of p53 at Ser15 after 30 min (350.7 ± 27.3%) and 60 min (256 ± 30.1%) that was not observed in presence of JNK inhibitor (118.2 ± 10.8% and 115.6 ± 16.7%, respectively).

These results were also corroborated by immunocytochemistry. Oligodendrocytes were treated with AMPA plus CTZ, in the absence or presence of 1 μM SP600125 (Figures 9B,C). Changes in the total expression of p53, associated with its activation, were determined using the p53 antibody recognizing total p53 and the presence of phosphorylation in Ser15 was detected by the specific antibody p-p53 (Ser15). As shown in Figure 9B, cells treated with AMPA displayed p53 activation (green) which was not observed in control and AMPA plus SP600125-treated cells. Additionally, JNK inhibition prevented AMPA-induced phosphorylation of p53 at Ser15 and therefore, its location in the cellular areas stained with the COX-IV antibody was diminished (Figure 9C).

On the other hand, our co-immunoprecipitation experiments support a direct interaction between CK2 and p53. For that, and in order to check the possible role of CK2 in the phosphorylation of p53 at Se15 and its translocation into mitochondria, we activated AMPA receptors in oligodendrocytes in presence of CK2 inhibitor TBB and analyzed by immunocytochemistry the expression of p53 (Figure 9B) and p-p53 (Ser15; Figure 9C). The results indicated that CK2 inhibition reduced both total p53 and p-p53 (Ser15) expression triggered by AMPA, as well as its mitochondrial accumulation.

These results suggest a sequential activation of the CK2/JNK/p53 axis triggered by the overstimulation of AMPA receptors in oligodendrocytes, which will lead to cell death by apoptosis.

## Discussion

As oligodendrocytes are highly vulnerable to overactivation of GluRs, oligodendroglial excitotoxicity may be considered a major factor in demyelinating disorders of the CNS like MS disease. Under pathologic conditions, several components of the glutamate system are altered, compromising glutamate homeostasis. Proinflammatory cytokines (e.g., TNFα and IL-1β) negatively regulate glutamate transporter expression and activity of, raising extracellular glutamate concentrations (Tilleux and Hermans, 2007). In turn, immune activation increases cystine-glutamate exchanger (xCT) expression that may result in higher glutamate release and excitotoxic damage to oligodendrocytes (Domercq et al., 2007; Pampliega et al., 2011).

Together, these and other alterations cause an increase in extracellular levels of glutamate that mediates the overactivation of Ca^2+^-permeable GluRs in oligodendrocytes and contributes to tissue damage in demyelinating diseases and other oligodendropathies. Indeed, AMPA and kainate receptor antagonists ameliorate neurological symptoms in several forms of EAE used to model various stages of MS (Bolton and Paul, 2006; Matute et al., 2001; Matute, 2011).

The apoptotic program activated by AMPA receptor-mediated excitotoxic insults in oligodendrocytes has not been fully elucidated, and knowing it in detail could provide the necessary tools to develop effective protective therapies.

The results reported here show that apoptosis induced by excitotoxicity in oligodendrocytes is mediated by a JNK and p38 signaling cascade in a CK2-dependent manner. In addition, we found that p53 is activated in response to excitotoxic insults initiated by AMPA receptors, and that this activation is a downstream consequence of, at least, CK2 and JNK proapoptotic contribution. These data provide novel targets to limit oligodendroglial cell death triggered by glutamate excitotoxicity.

On the basis that CK2 is generally part of main prosurvival signaling pathways (Turowec et al., 2010; Zheng et al., 2011; Gray et al., 2014) our initial hypothesis was that CK2 could limit deleterious consequences of AMPA receptor activation in oligodendrocytes. Unexpectedly, AMPA excitotoxic insults trigger an increase in CK2 activity and both pharmacological inhibition and CK2 gene silencing attenuates toxicity induced by AMPA receptors stimulation in oligodendrocytes, concomitant with the interruption of the cascade of events leading to mitochondrial dysfunction. Thus, CK2 inhibition in oligodendrocytes attenuated depolarization of the mitochondrial membrane, which is an early event in apoptosis (for review see Atlante et al., 2001; Skulachev, 2006), as well as it reduced oxidative stress. These observations indicate that CK2 is required as part of the early apoptotic machinery that distort mitochondrial function in response to excitotoxic insults to oligodendrocytes, and suggest a proapoptotic role for CK2 in the regulation of oligodendrocyte cell death. Consistent with our results, renal ischemia/reperfusion injury results in increased CK2α expression, and CK2 inhibition attenuated inflammation and oxidative stress which results in reduced apoptosis and concomitant renal dysfunction (Ka et al., 2015). Conversely, reduced CK2 expression levels and lower total activity of this kinase, after mice cerebral ischemic injury, facilitates ROS production and mediates neuronal cell death (Kim et al., 2009).

Our findings indicate that CK2 regulates a central proapoptotic signaling pathway activated upon AMPA receptor stimulation. Along this line, CK2 can have a pro-apoptotic role in cellular events that involve JNK activation and CK2-JNK interactions (Min et al., 2003; Hilgard et al., 2004). JNK are important stress-responsive kinases that are activated by various forms of insults, including ischemia (Nijboer et al., 2011) and glutamate excitotoxicity (Shah et al., 2014). Additionally, JNK signaling is involved in neuroinflammation, blood barrier disruption and oligodendroglial apoptosis in white matter injury (Wang et al., 2012). In the current study, we observed that JNK signaling pathway activated by AMPA receptor stimulation involves the joint activation of p38 pathway. This phenomenon presumably occurs via ASK1, a MAPK kinase that activates downstream MAPKs, JNK and p38, and regulates neuroinflammation and demyelination by TLR-ASK1-p38 pathway (Guo et al., 2010). In line with this idea, we have recently observed that ASK1 pharmacological inhibition with NQDI reduces AMPA-induced oligodendrocyte cell death and that this protection is based on the ability to inhibit downstream JNK and p38 activation (data not shown).

In the current study, we have also found a link between the activation of CK2, JNK and p38. Thus, CK2 pharmacological inhibition with TBB jointly reduces JNK and p38 activation after AMPA excitotoxic insult. This suggests that CK2 acts as a link between early stress events induced by AMPA excitotoxicity in oligodendrocytes and pro-apoptotic activation of hypothetical JNK/p38 axis.

The tumor suppressor transcription factor p53 plays a central role in induction of cell-cycle arrest, senescence, and apoptosis in the response to a variety of prolonged or severe stresses (Loughery and Meek, 2013) and a number of p53-modifying enzymes have been identified, for example MAPK p38 and JNK (Wu, 2004; Cargnello and Roux, 2012). The link between JNK/p38 activation and p53 apoptotic contribution has also been previously described in glutamatergic excitotoxicity conditions in hippocampal neurons and differentiated PC12 cells (Choi et al., 2011; Jiang et al., 2014). Thus, knock-down or blockade of JNK inhibited glutamate-induced p53 activation in that excitotoxic paradigm (Choi et al., 2011). In the present study, we observed that p53 may be one of the downstream consequences of CK2/JNK signaling pathway activation, since both CK2 or JNK inhibition were able to limit p53 activation/stabilization status induced by AMPA excitotoxic insult. This suggests that in oligodendrocyte excitotoxicity CK2 mediates p53 activation through JNK phosphorylation. Ser15 phosphorylation is required for p53 function in the physiological context of p53-responsive promoters that suggests a key role even for low levels of this modification in promoting p53-transcription function (Loughery et al., 2014). Accordance with this, we propose the modulation of p53 phosphorylation at the Ser15 through CK2/JNK as an essential step in oligodendrocyte apoptotic death, alone or in conjunction with p38, as described in previous reports (Gong et al., 2010; Fausti et al., 2013).

So, the fact that both CK2 and JNK inhibition mediated reduction in mitochondrial phospho-p53 (Ser15) could be indicating that these kinases are the mediators of a mitochondrial specific p53 modification implicated in AMPA-induced oligodendrocyte cell death.

On the other hand, our co-immunoprecipitation experiments support a direct interaction between CK2 and p53. In this sense, CK2 in a complex with the chromatin factor FACT (hSPT16 and SSRP1), was identified as the kinase responsible for p53 phosphorylation at Ser392 after UV exposure *in vitro* (Keller et al., 2001; Finlan et al., 2006). Thus, CK2 catalytic activity towards p53 is enhanced by FACT *in vitro*, and Ser392 phosphorylation leads to an increase in p53 DNA binding and transcriptional activity in cells (Keller et al., 2001; Keller and Lu, 2002). Moreover, it has been described that CK2 is implicated in the response to UV irradiation-induced DNA damage, regulating p53 tumor suppressor protein functions through phosphorylation of Ser392 in p53 (Cox and Meek, 2010). In our excitotoxic context, we have described that AMPA triggers the molecular interaction between CK2 and p53, induces the translocation of p53 into the nucleus and that CK2 inhibitor reduces these effects (Figure 7). So, we were also trying to detect p-p53 (Ser392) in oligodendrocytes under excitotoxicity and a putative link between CK2 and p-p53 (Ser392) in this context. However, we have not observed p53 phosphorylation on Ser392 residue in oligodendrocytes after AMPA treatment (data not shown), so that p-p53 (Ser392) may in fact distinguish AMPA excitoxicity from other stimuli that have been described as having p53 activation through phosphorylation of Ser392.

The signaling described here raises the question of how a kinase like CK2, *a priori* prosurvival, may contribute to apoptotic program triggered by AMPA excitotoxic insult in oligodendrocytes. In this regard, JNK has been proposed as mediator of CK2 proapoptotic activity (Min et al., 2003), however the molecular mechanism through which CK2 can connect with JNK/p38 apoptotic pathway, and even with ASK1, under AMPA excitotoxic conditions needs to be explored in more detail. We hypothesize that CK2 is activated during early stages of apoptosis modulating a signaling mechanism with a dual action (anti or pro-apoptotic), which is able to influence a hypothetical ASK1/JNK/p38 axis. The UPR and, specifically, the IRE1α pathway, could be the link between CK2 and pro-apoptotic JNK/p38 activation.

CK2 is also a regulator of the inflammatory response (Singh and Ramji, 2008), an intrinsic process that underlies many neurodegenerative diseases. Specifically, CK2 modulates neuroinflammatory events characteristic of Alzheimer’s disease (Rosenberger et al., 2016) and experimental autoinmmune encephalomyelitis (Axtell et al., 2006; Sestero et al., 2012; Gibson et al., 2017). Our results indicate that CK2 could be a therapeutic target not only restricted to the inflammatory process but also in relation to apoptotic program that triggers oligodendrocyte death in MS.

In summary, the findings reported here indicate that CK2 inhibition exerts oligoprotective effects from excitotoxic conditions induced by moderate activation of AMPA receptors, as it reduces ROS production and loss in mitochondrial potential. In turn, this cytoprotective effect of CK2 was mediated by a decrease in pro-apoptotic activation of JNK/p38/p53 signaling axis induced by AMPA receptors stimulation. We propose that CK2 inhibition is a promising target to protect oligodendrocytes from AMPA induced excitotoxic insults with potential implications for the treatment of demyelinating pathologies, especially MS disease.

## Author Contributions

MC-A, CM and MS-G conceived and designed the experiments and analyzed data. MC-A was involved in all experiments. MS participated in experiments with optic nerves of transgenic mice, their quantification and analysis of data. AM and SM took part in qPCR and subsequent analysis. AR, FL and JZ helped in the *in vitro* kinase assays and gene silencing experiments and interpreted the results. MS, FP-C and MS-G performed immunocytochemical analysis. MC-A, CM and MS-G prepared and wrote the manuscript and all authors provided critical feedback on the manuscript drafting and approved the final version to be published.

## Conflict of Interest Statement

The authors declare that the research was conducted in the absence of any commercial or financial relationships that could be construed as a potential conflict of interest.
